# Feature Fusion of Deep Spatial Features and Handcrafted Spatiotemporal Features for Human Action Recognition

**DOI:** 10.3390/s19071599

**Published:** 2019-04-02

**Authors:** Md Azher Uddin, Young-Koo Lee

**Affiliations:** Department of Computer Science and Engineering, Kyung Hee University, Global Campus, Yongin 17104, Korea; azher006@yahoo.com

**Keywords:** deep spatial features, spatiotemporal features, Inception-Resnet-v2, Weber’s law based volume local gradient ternary pattern

## Abstract

Human action recognition plays a significant part in the research community due to its emerging applications. A variety of approaches have been proposed to resolve this problem, however, several issues still need to be addressed. In action recognition, effectively extracting and aggregating the spatial-temporal information plays a vital role to describe a video. In this research, we propose a novel approach to recognize human actions by considering both deep spatial features and handcrafted spatiotemporal features. Firstly, we extract the deep spatial features by employing a state-of-the-art deep convolutional network, namely Inception-Resnet-v2. Secondly, we introduce a novel handcrafted feature descriptor, namely Weber’s law based Volume Local Gradient Ternary Pattern (WVLGTP), which brings out the spatiotemporal features. It also considers the shape information by using gradient operation. Furthermore, Weber’s law based threshold value and the ternary pattern based on an adaptive local threshold is presented to effectively handle the noisy center pixel value. Besides, a multi-resolution approach for WVLGTP based on an averaging scheme is also presented. Afterward, both these extracted features are concatenated and feed to the Support Vector Machine to perform the classification. Lastly, the extensive experimental analysis shows that our proposed method outperforms state-of-the-art approaches in terms of accuracy.

## 1. Introduction

Human action recognition is an attractive research topic in the area of computer vision due to its wide range of applications in video surveillance, sports video analysis, movie search, etc. Action recognition is challenging due to different viewpoint, occlusions, clothing, and the subject’s appearance, personal style, action length, and complex background motion [1,2,3,4]. Despite extensive research done on this topic, several issues still need to be resolved.

Feature extraction is an essential and core step for image and video analysis [1,2,4,5,6,7,8,9,10]. Basically, there are two different kinds of feature descriptor for the video representation, one is hand-crafted descriptors and another one is deep-learning based descriptors. The deep-learning based approach determines the trainable feature automatically from the video, whereas handcrafted descriptors obtain the features based on the manually designed algorithms. Recently, the Convolutional Neural Networks (CNN) based approach is the most commonly studied approach in numerous disciplines of computer vision and image processing, including action classification, and has obtained an excellent achievement. CNN is basically practiced to obtain spatial features from the static image. On the other hand, Wang et al. [11] presented that motion information obtained through HOF descriptor is adequate to produce a satisfactory classification result for the action recognition problem, however, it is not sufficient to fully specify an action, particularly when actions are actively associated with particular objects. Conversely, single frame based CNN is able to extract the spatial information from the video sequence, which is very important to represent the appearance, however, they failed to extract the motion information [12,13]. Furthermore, a two-stream CNN architecture [10] by combining spatial and optical based motion information is also introduced. However, optical flow is the apparent motion of intensity values, which can be produced by lighting changes without any actual motion.

Previously, in [1,2,3,7,14] texture based spatiotemporal features are introduced to resolve the problem of action recognition due to their notable achievements and computational efficiency. However, these approaches are variants of Local Binary Pattern (LBP) [6] and suffer from similar kinds of issues that LBP faces, which include sensitivity to noise and limited capability to obtain more discriminative information. Moreover, these approaches do not take shape information into account. In order to address the above-mentioned issues in this paper, we introduce a novel approach to recognize the human actions by considering both deep spatial features and handcrafted spatiotemporal features. At first, we extract the deep spatial features by employing a state-of-the-art deep convolutional network, namely Inception-Resnet-v2 [15]. The Inception-Resnet-v2 net is responsible to extract the local features from each frame. Later on, these local features are aggregated to form the global features of each video. In parallel, we also apply our proposed dynamic feature descriptor, Weber’s law based Volume Local Gradient Ternary Pattern (WVLGTP) to bring out the effective spatiotemporal features. Furthermore, a multi-resolution strategy for WVLGTP based on an averaging scheme is also introduced. Afterward, both these features (deep spatial features and handcrafted spatiotemporal features) are concatenated and feed to the Support Vector Machine (SVM) [16] to achieve the classification. To evaluate the performance of our work, five benchmark datasets are employed, including the KTH dataset [17], UCF Sports action dataset [18,19], UT-Interaction dataset [20], Hollywood2 [21], and UCF-101 dataset [22]. The key contribution of this work is summarized as follows:

- In this work, we propose a novel approach for human action recognition by fusing deep spatial feature and handcrafted spatiotemporal feature.
- We introduce a novel handcrafted feature descriptor, namely Weber’s law based Volume Local Gradient Ternary Pattern (WVLGTP), which brings out the spatiotemporal features. It also takes shape information into account by using gradient operation. Furthermore, Weber’s law based threshold value and the ternary pattern based on an adaptive local threshold is introduced to effectively handle the noisy center pixel value.
- Besides, a multi-resolution approach for WVLGTP based on an averaging scheme is also presented.
- Lastly, we present an extensive experimental analysis to prove the effectiveness of our approach over state-of-the-art.

The remainder of the paper is represented as follows. Section 2 surveys related researches, while Section 3 thoroughly describes the proposed approach for action recognition. Datasets and experimental results are explained in Section 4. In the end, conclusions are drawn in Section 5.

## 2. Related Work

Numerous researches have been proposed in the area of action recognition. However, mainly two different types of descriptors are studied in the existing works: hand-crafted descriptors and deep learning based descriptors. In this section, we will discuss the related approaches those are most relevant to our work.

### 2.1. Hand-Crafted Descriptor

Baumann et al. [1] introduced Volume Local Binary Pattern (VLBP) for action recognition, which was first proposed in [5] for recognizing the facial expressions. VLBP describes the dynamic texture feature by comparing the intensity values of the neighboring voxels in the spatiotemporal domain. Later on, Baumann et al. [2] also proposed the Motion Binary Pattern (MBP), which extracts the motion feature to recognize the actions. However, these approaches sensitive to noise and suffers from the illumination problem, since they are the simple extension of Local Binary Pattern (LBP) [6]. Similar to [1,2], Uddin et al. [7] presented Adaptive Local Motion Descriptor (ALMD) to recognize the human actions and their approach produced consistent patterns against intensity fluctuation due to employing adaptive ternary pattern concept. Besides, extended computation of LBP to 9 slices for three orthogonal planes are introduced in [14] to recognize the human actions. Moreover, here the authors employed the bag of words recognition model after detecting the space-time interest points. Furthermore, Laptev et al. [3] proposed Local Ternary Pattern for three orthogonal planes (LTP-TOP) in order to perform the action recognition. Local Trinary Patterns based on comparing nearby patches are proposed in [23], which is robust to variations in texture. In addition, Guo et al. [24] introduced 3D gradient LBP based feature descriptor, which takes the benefit of the neighborhood information of cuboids in three dimensions. A novel Salient Foreground Trajectory (SFT) based extraction method is introduced in [4] by employing background trajectory subtraction to represent the trajectory-based feature for action recognition. In [8], an unsupervised approach is proposed that models the action as mid-level action elements (MAE) in hierarchical structure. Lastly, Tu et al. [25] presented a novel Multi-label Hierarchical Dirichlet Process (ML-HDP) to recognize the actions by proposing a generative topic model and utilizing the iDT (Improved Dense Trajectories) [9] with MBH (Motion Boundary Histogram) [26] descriptor, which simultaneously represents multiple complex movements and motion segments at various hierarchical levels. Previously, wang et al. [27] introduced the Dense Trajectories (DT) for video representation, which captures the foreground local motion. Low-level feature-based approaches were introduced in [28,29] respectively. Ohnishi et al. [30] presented a novel low-level feature descriptor that pools crossed convolutional layers with iDT.

### 2.2. Deep Learning Based Descriptor

Deep learning based methods bring out the optimal features automatically from the video data. Simonyan et al. [10] proposed the deep learning based approach for recognizing the human actions, in which spatial information is obtained by spatial ConvNet and dynamic information is obtained by multi-frame dense optical flow with ConvNet. However, optical flow is the apparent motion of intensity patterns, which can also be produced by lighting variations without any actual motion and may lead to wrong information. Similarly, Karpathy et al. [12] introduced a multi-resolution CNN structure for large-scale action recognition, in which they utilized low-resolution stream and high-resolution stream. However, they did not consider the motion information. Later on, in [31] the trajectory-based feature with CNN is employed to extract the features from the video data to classify the actions. Similar to [31], Lu et al. [32] also proposed trajectory pooling approach along with 3D ConvNets for action recognition, in which they computed multiscale dense trajectories and on 3D ConvNets they produced trajectory pooling. In [33], the authors extracted the features in multiple temporal scales and employed Res3D neural network model. Furthermore, they acquired information from RGB channels and optical flow. Wang et al. [34] introduced a deep multi-stream neural network using ResNet architecture that fuses temporal awareness for action recognition. Here, they employed RGB video frame, optical flow and warped optical flow images as input to ResNet. ActionVLAD CNN layer is introduced in [35], which aggregates both the spatial and temporal features. However, they lose much temporal features in the sequential frames. Later on, Sequential Video VLAD layer is proposed in [36], which addresses the issues of ActionVLAD. Zhao et al. [37] introduced an efficient pooling approach called Line pooling, which pools stacked features along the timeline.

## 3. Proposed Method

**Figure 1:**
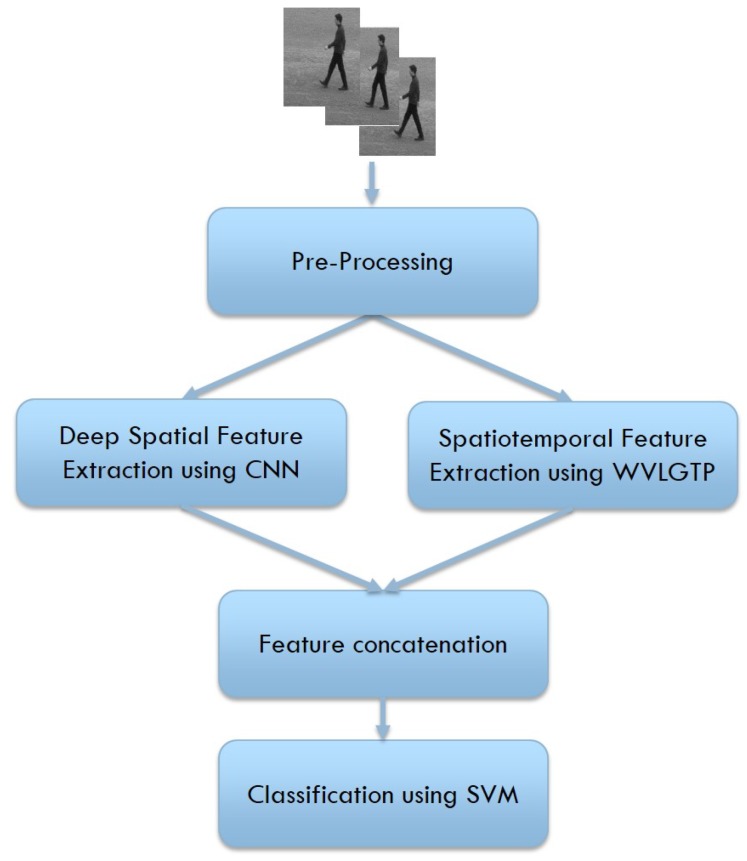
Flowchart of our proposed approach for human action recognition.

In this section, we describe our proposed approach for recognizing human actions. At first, we perform few pre-processing operations that include frame extraction and frame resizing. Afterward, our proposed novel feature descriptor, namely Weber law based Volume Local Gradient Ternary Patterns (WVLGTP) is employed to extract the significant spatiotemporal information from the videos. In parallel, we also applied the Inception-Resnet-v2 network [15] to bring out the deep spatial features from the videos. Later on, these features are concatenated and feed to the Support Vector Machine (SVM) [16] to perform the classification. Figure 1 demonstrates the proposed approach to recognize the actions.

### 3.1. Deep Spatial Feature Extraction Using Inception-Resnet-v2 Network

**Figure 2:**
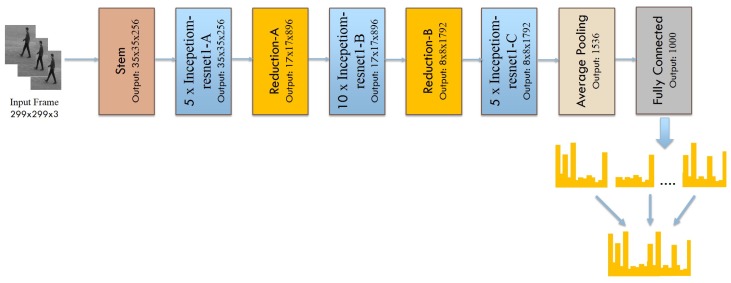
Deep spatial feature extraction using the Inception-Resnet-v2 network.

In order to obtain the deep spatial features from the videos, we adopt the state-of-the-art Inception-Resnet-v2 network [15], which is a combination of two recent architectures, one is Residual connections [38] and another one is Inception architecture [39]. The employed Inception-Resnet-v2 model includes Stem, Inception Resnet, and Reduction layers. These layers are followed by an average-pooling layer and a fully connected layer with 1000 channels. The Stem includes preliminary convolution operations executed before entering the Inception blocks. The Inception Resnet layers include residual connections along with the convolution operation whereas Reduction layers are responsible for adjusting the height and width of the grid. The convolutional layers are responsible for extracting the spatial features while pooling layers decrease the dimensionality of individual feature map, but hold the most significant features and make the model invariant to illumination and translation. Furthermore, the convolutional layers are followed by the batch normalization layer and Rectified Linear Unit (ReLU), which is a nonlinearity function and helped to decrease the training time. The Inception-Resnet-v2 takes individual RGB frames as input with size 299×299×3, which captures the spatial information from each video frame. The appearance information represented by spatial information is a very important clue because many actions are actively correlated with particular objects. Figure 2 illustrates the architecture of deep spatial feature extraction using Inception-Resnet-v2 network. The Inception-Resnet-v2-Net extracts the local features from each frame. Later on, these local features are aggregated to form the global features of each video.

### 3.2. Spatiotemporal Feature Extraction Using WVLGTP

**Figure 3:**
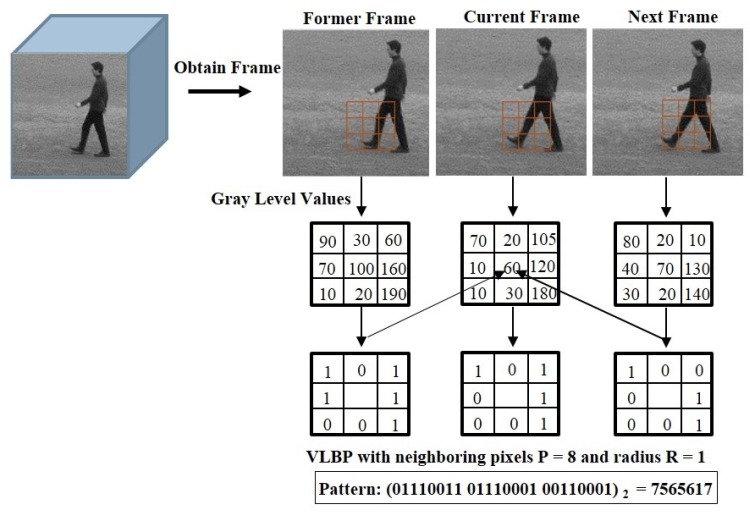
An approach of computing Volume Local Binary Pattern (VLBP) on three successive frames.

**Figure 4:**
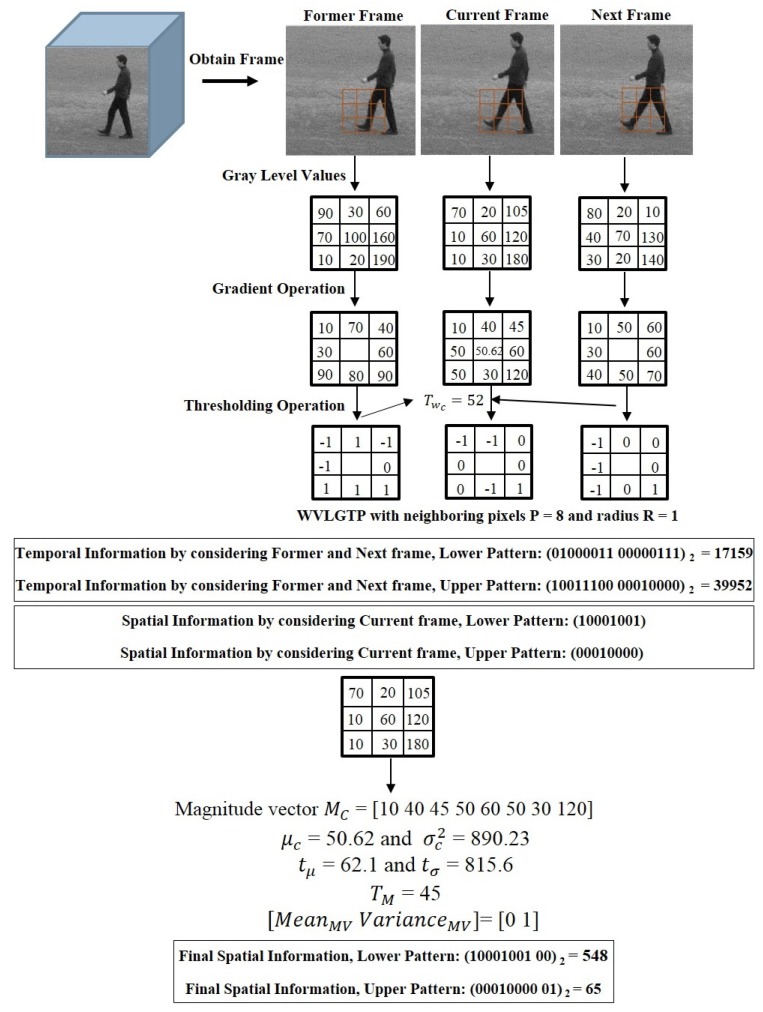
The feature extraction process of Weber’s law based Volume Local Gradient Ternary Pattern (WVLGTP).

To improve the performance of spatiotemporal feature descriptor, we introduce Weber’s law based Volume Local Gradient Ternary Pattern (WVLGTP), which represent both spatial and dynamic information. The basic spatiotemporal feature descriptor Volume Local Binary Pattern (VLBP) [1] works similar to the conventional LBP (Local Binary Pattern) [6], which compares the gray value of neighboring voxels within the space-time volume of the voxel’s center and assign 1 or 0 accordingly. Figure 3 illustrates the approach of calculating VLBP on three successive frames. Hence, VLBP also suffers from a similar type of problems that LBP undergoes. In order to extract the information more effectively, we proposed WVLGTP, which brings out the shape information using gradient operation. Furthermore, Weber’s law based threshold value and the ternary pattern based on an adaptive local threshold is introduced to effectively handle the noisy center pixel value. Besides, a multi-resolution approach for WVLGTP based on an averaging scheme is presented, which is able to tackle the illumination problem. Figure 4 explains the process of WVLGTP in three consecutive frames. At first, gradient operation is performed on the consecutive frames by computing the absolute difference between the neighbor pixels and given center pixel, which represent the shape information. Here, we compute the gradient for neighboring pixels $C_{n}$ of the center frame *C* as $g_{C_{n}}=\left|C_{n}-C_{c}\right|$, where $C_{c}$ is the center frame center pixel value and then, set the center frame center pixel value as ${\bar{g}}_{C_{c}}=\frac{1}{l}\sum_{n=0}^{l-1}g_{C_{n}}$ by taking the average of *l* neighboring gradient values. Similarly, the gradient for neighboring pixels $F_{n}$ of the former frame *F* and the gradient for neighboring pixels $N_{n}$ of the next frame *N* are computed as $g_{F_{n}}=\left|F_{n}-F_{c}\right|$ and $g_{N_{n}}=\left|N_{n}-N_{c}\right|$, respectively. Furthermore, weber’s law based threshold is introduced to effectively handle the noisy center pixel value. Weber’s Law is a psychological law [40], which can be expressed as

(1) $$\frac{\Delta I}{I}=K$$

where ΔI denotes the increment threshold which represents the noticeable difference for discrimination; *I* denotes the initial intensity, and *K* denotes the constant. Previously, Weber Local Descriptor was introduced in [41] to extract the image (spatial) features effectively.

In our work, weber’s law based threshold value $T_{w_{c}}$ is employed to effectively compute the spatiotemporal features, which is defined as

(2) $$T_{w_{c}}={\bar{g}}_{C_{c}}+\sum_{n=0}^{l-1}\frac{|C_{n}-C_{c}|}{C_{c}}$$

here, $\frac{|C_{n}-C_{c}|}{C_{c}}$ denotes the Weber fraction. To generate the spatiotemporal feature vector FV, Weber’s law based threshold value $T_{w_{c}}$ is subtracted from the neighboring gradient values of the former frame *F*, current frame *C*, and next frame *N* which are expressed by the following equations,

(3) $$WVLGTP\_F_{P,R}\left(x,y\right)=S\left(g_{F_{n}}-T_{w_{c}}\right)$$

(4) $$WVLGTP\_C_{P,R}\left(x,y\right)=S\left(g_{C_{n}}-T_{w_{c}}\right)$$

(5) $$WVLGTP\_N_{P,R}\left(x,y\right)=S\left(g_{N_{n}}-T_{w_{c}}\right)$$

(6) $$Where,S\left(x\right)=\left\{\begin{array}{cc} 1, & ifx>T\\ -1, & ifx<-T\\ 0, & otherwise \end{array}\right.$$

here, adaptive local threshold *T* is derived to convert the intermediate features into ternary codes and (P,R) represents the number of neighboring pixels and radius respectively. *T* is computed by taking the median of $|g_{C_{n}}-T_{w_{c}}|$. In our example, *T* equals to 8. In [1,5], VLBP takes 8 neighboring pixels from three continuous frames, which generates a huge feature vector and leads to ambiguities. In order to overcome this issue, in our work we consider the spatial information and temporal information separately. The feature vector for temporal information is produced by comparing the former frame and next frame with the current frame, whereas the feature vector for spatial information is produced based on the current frame. Furthermore, the magnitude vector $M_{C}$ is also considered during the generation of spatial features. The magnitude vector acts as an auxiliary feature and includes more discriminating power. In this work, we introduce two one-dimensional features of the mean and the variance of $M_{C}$ to keep the magnitude information rather than directly utilizing the magnitude vector. The mean of the magnitude vector indicates its average deviation and the variance of magnitude vector indicates its overall changes. The mean $\mu_{c}$ and variance $\sigma_{c}^{2}$ of the magnitude vector is computed based on the following equations

(7) $$\mu_{c}=\frac{1}{l}\sum_{n=0}^{l-1}g_{C_{n}}$$

(8) $$\sigma_{c}^{2}=\frac{1}{l}\sum_{n=0}^{l-1}{\left(g_{C_{n}}-\mu_{c}\right)}^{2}$$

(9) $$Mean_{MV}=S_{M}\left(\mu_{c}-t_{\mu }\right)$$

(10) $$Variance_{MV}=S_{M}\left(\sigma_{c}^{2}-t_{\sigma }\right)$$

(11) $$Where,S_{M}\left(x\right)=\left\{\begin{array}{cc} 1, & ifx>T_{M}\\ -1, & ifx<-T_{M}\\ 0, & otherwise \end{array}\right.$$

where local thresholds $t_{\mu }$ and $t_{\sigma }$ are the average values of mean $\mu_{c}$ and variance $\sigma_{c}^{2}$ in the current sub-frame. Here, $T_{M}$ is derived to convert the magnitude vector into ternary codes. $T_{M}$ is also computed by taking the median of $|C_{n}-C_{c}|$. Later on, the feature vectors for spatial information and temporal information is concatenated for both lower and upper patterns.

#### Multi-Resolution Approach for WVLGTP

**Figure 5:**
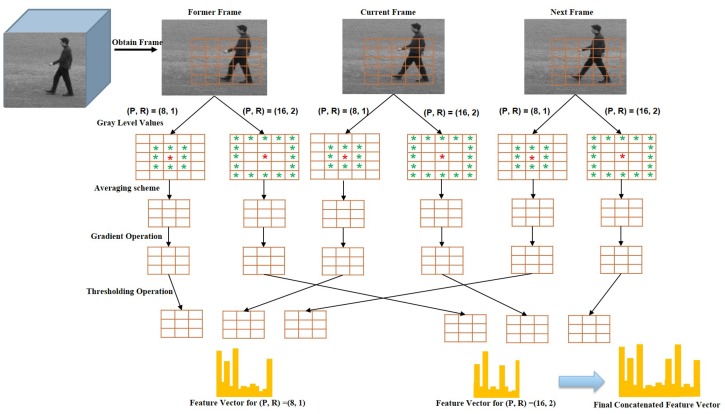
The Multi-resolution approach of WVLGTP.

**Figure 6:**
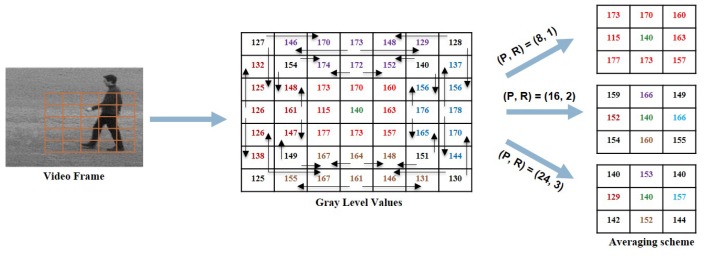
Averaging scheme for the multi-resolution approach.

Above we present the approach of extracting WVLGTP on a single scale with the number of neighboring pixels P=8 and radius R=1. In this work, a multi-resolution approach for the proposed descriptor is applied to increase the recognition accuracy. The multi-scale WVLGTP feature vector is formed by combining the WVLGTP feature vector of every single resolution with varying *P* and *R*. Figure 5 demonstrates the multi-resolution approach of WVLGTP. Furthermore, in this work, we also introduced an averaging scheme before performing the gradient operation, which is able to tackle the illumination problem. In this averaging scheme, it computes the average of neighboring pixels and forms new intensity value. Figure 6 shows the process of the averaging scheme for the multi-resolution approach, in which direction and color represent the neighboring pixels used for computing the average value. For example, with P=16 and radius R=2, the resultant intensity value 149 of the top right pixel (i+1,j−1) is computed by taking average of $\left\{152,140,156\right\}$.

### 3.3. Classification Using SVM

Afterward, the deep spatial features generated by the Inception-Resnet-v2 network and spatiotemporal features produced by WVLGTP are concatenated and fed into a Support Vector Machine (SVM) for classification. In our work, we employ nonlinear SVM with the RBF kernel function [16] to classify the actions from the feature vector. The best parameters *C* and γ were selected by performing 5-fold cross-validation.

## 4. Experiments

In this part, we evaluate the proposed approach on five benchmark datasets, which include the KTH dataset [17], UCF Sports action dataset [18,19], UT-Interaction dataset [20], Hollywood2 [21], and UCF-101 dataset [22]. The UCF-101 dataset consists of a large number of video clips with many action categories, while the UT-Interaction dataset represents the interaction between two persons. In contrast, the Hollywood2 dataset represents complex activities rather than simple actions like the KTH dataset. Furthermore, Hollywood2 is quite a challenging dataset since each video includes notable camera motion and fast scene changes. At first, we present the datasets and implementation details. Later on, experimental analysis and the comparisons with state-of-the-art approaches are demonstrated. In our work, performance is measured by the average accuracy.

### 4.1. KTH Dataset

**Figure 7:**
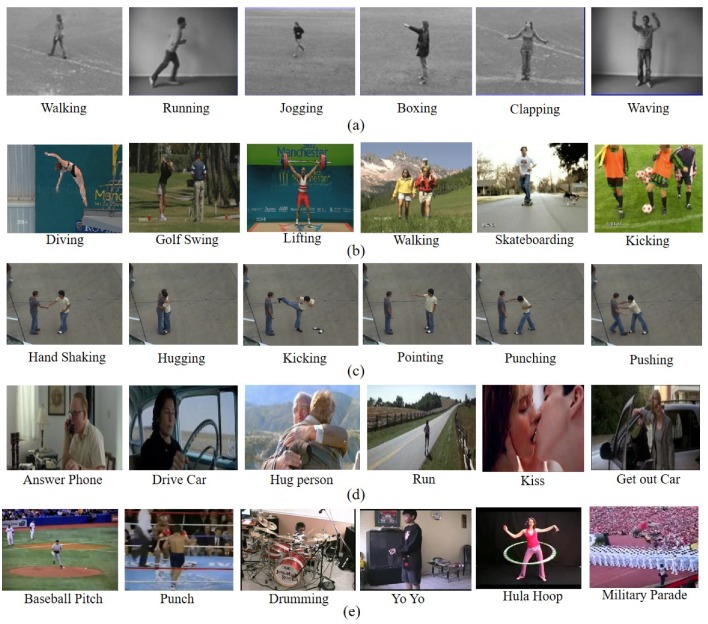
(**a**) Sample frames of the KTH dataset; (**b**) Sample frames of the UCF Sports action dataset; (**c**) Sample frames of the UT-Interaction dataset; (**d**) Sample frames of the Hollywood2 dataset, and (**e**) Sample frames of the UCF-101 dataset.

The KTH dataset [17] comprises 600 videos including six action categories: walking, running, jogging, boxing, clapping, and waving. Each action includes 100 sequences performed by 25 persons in four diverse situations. Sample frames of KTH dataset are presented in Figure 7a.

### 4.2. UCF Sports Action Dataset

The UCF Sports action dataset [18,19] includes 150 video sequences with a resolution of 720×480. It comprises 10 actions that include walking, running, kicking, lifting, diving, golf swing, riding horse, skateboarding, swing-side, and swing-bench. These actions are performed in different real environments that cover diverse viewpoints and also including a lot of camera motion. An instance of UCF Sports action dataset is displayed in Figure 7b.

### 4.3. UT-Interaction Dataset

The UT-Interaction dataset [20] consists of 120 videos with a resolution of 720×480. This dataset includes 6 action classes: shake-handing, hugging, kicking, pushing, pointing, and punching. Several persons with 15 different clothing conditions do these actions. An example of the UT-Interaction dataset is presented in Figure 7c.

### 4.4. Hollywood2 Dataset

The Hollywood2 dataset [21] consists of 3669 video clips with 12 classes of human actions. The actions include answer phone, drive car, eat, fight person, get out car, hand shake, hug person, kiss, run, sit down, sit up, and stand up. Hollywood2 is a very challenging dataset since each video clip includes notable camera motion and rapid scene switches. Figure 7d shows some sample frames of the Hollywood2 dataset.

### 4.5. UCF-101 Dataset

The UCF-101 dataset [22] is one of the largest action datasets, which consists of 13,320 video clips including 101 action classes. All of these videos are obtained from YouTube. The videos are divided into 25 groups covering 4 to 7 action sequences from each group. An example of the UCF-101 dataset is presented in Figure 7e.

### 4.6. Model Training and Testing

For all the five datasets, 70 percent of the video data is used for training and the remaining 30 percent are applied during the testing. Furthermore, we also employed the 5-fold cross-validation approach. In our work, we trained the Inception-Resnet-v2 network on Matlab R2018b. Similar to previous works [10,32,34], we initialized the parameters of Inception-Resnet-v2 from a pre-trained ImageNet model. The stochastic gradient descent (SGD) algorithm is employed with a mini-batch size of 128 and the momentum set to 0.9. Furthermore, during the training phase, we employed data augmentation by performing the horizontal reflection of the video frames to reduce overfitting [42]. During testing, we sample a fixed number of video frames (set to 35) with same temporal spacing among them [10,34].

### 4.7. Experimental Analysis

**Figure 8:**
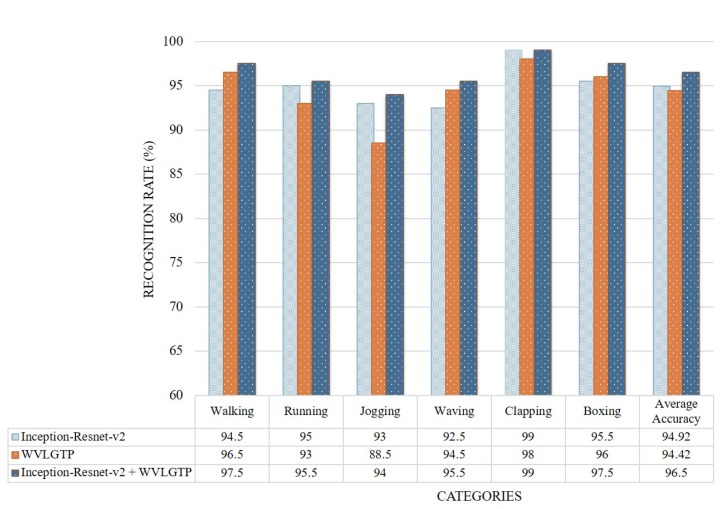
Accuracy comparison of each class by Inception-Resnet-v2, WVLGTP, and Inception-Resnet-v2 plus WVLGTP on the KTH dataset.

We performed various experiments to investigate the performance of the proposed approach to recognize human actions. Figure 8 presents the accuracy comparison of each category on the KTH dataset. This experiment shows that our proposed method performs best on clapping, boxing, and walking categories while running, jogging, and waving classes show competitive recognition rate. The average accuracy when employing Inception-Resnet-v2 network, WVLGTP and Inception-Resnet-v2 plus WVLGTP on the KTH dataset were 94.9%, 94.4%, and 96.5%, respectively.

**Figure 9:**
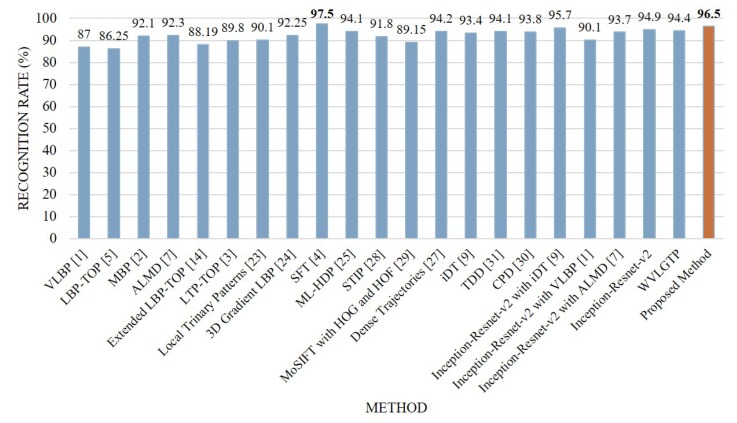
Comparison between the proposed method and other state-of-the-art approaches on the KTH dataset.

Comparison between the proposed approach and other state-of-the-art approaches on the KTH dataset is depicted in Figure 9. From this figure, we can see that the proposed descriptor, WVLGTP shows superior performance over the existing dynamic texture feature descriptors, which include VLBP [1], LBP-TOP [5], MBP [2], ALMD [7], Extended LBP-TOP [14], LTP-TOP [3], and 3D Gradient LBP [24]. Similarly, WVLGTP outperformed the STIP [28], MoSIFT [29], Dense Trajectories [27], iDT [9], TDD [31], and CPD [30] by 2.6%, 5.25%, 0.2%, 1%, 0.3%, and 0.6%, respectively. Furthermore, the proposed method (Deep spatial features with WVLGTP) also outperforms ML-HDP [25] by 2.4% on the KTH dataset. However, SFT [4] shows the best performance on KTH dataset and beats the proposed approach by 1%.

On the KTH dataset, our proposed method (Inception-resnet-v2 with WVLGTP) shows 96.5% accuracy, whereas Inception-resnet-v2 with iDT [9], Inception-resnet-v2 with VLBP [1], and Inception-resnet-v2 with ALMD [7] show 95.7%, 90.1%, and 93.7% accuracy, respectively.

**Figure 10:**
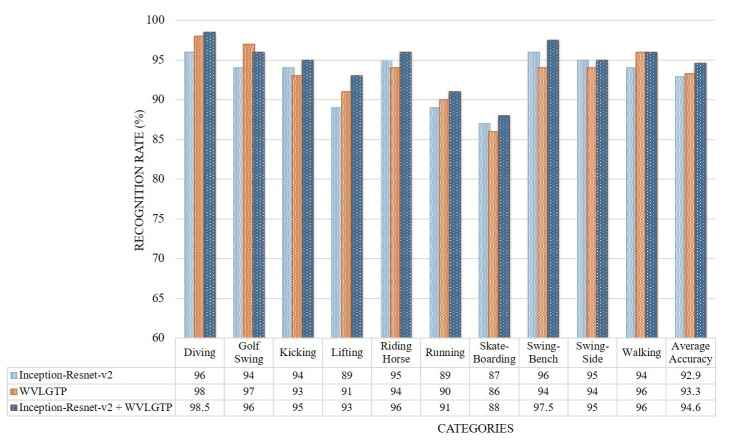
Accuracy comparison of each class by Inception-Resnet-v2, WVLGTP, and Inception-Resnet-v2 plus WVLGTP on the UCF sports action dataset.

**Figure 11:**
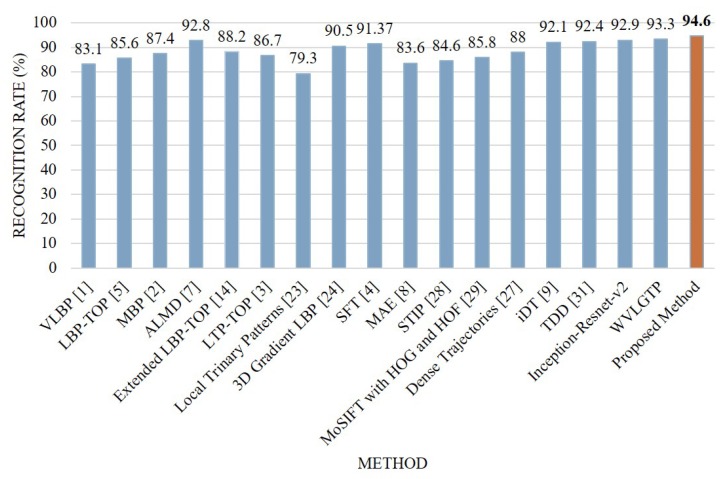
Comparison between the proposed method and other state-of-the-art approaches on the UCF sports action dataset.

Similar to the above experiments, Figure 10 demonstrates the accuracy comparison of each category by Inception-Resnet-v2, WVLGTP, and Inception-Resnet-v2 plus WVLGTP while Figure 11 presents the comparison between the proposed method and other state-of-the-art approaches on UCF sports action dataset. The average recognition rates when applying Inception-Resnet-v2 network, WVLGTP and Inception-Resnet-v2 plus WVLGTP on UCF sports action dataset were 92.9%, 93.3%, and 94.6%, respectively. For the UCF sports action dataset, diving, riding horse, and swing-bench classes show higher accuracy when applying the proposed approach while skateboarding shows lower recognition rate due to the complexity of skateboarding video clips. On this dataset, our proposed method slightly beats the SFT [4] by 3.23% and significantly outperforms the Mid-level Action Elements (MAE) [8] by 11%. Besides, the proposed descriptor, WVLGTP shows excellent recognition rate over the state-of-the-art dynamic texture feature descriptors. Similarly, WVLGTP beats the STIP [28], MoSIFT [29], Dense Trajectories [27], iDT [9], and TDD [31] by 8.7%, 7.5%, 5.3%, 1.2%, and 0.9%, respectively.

**Figure 12:**
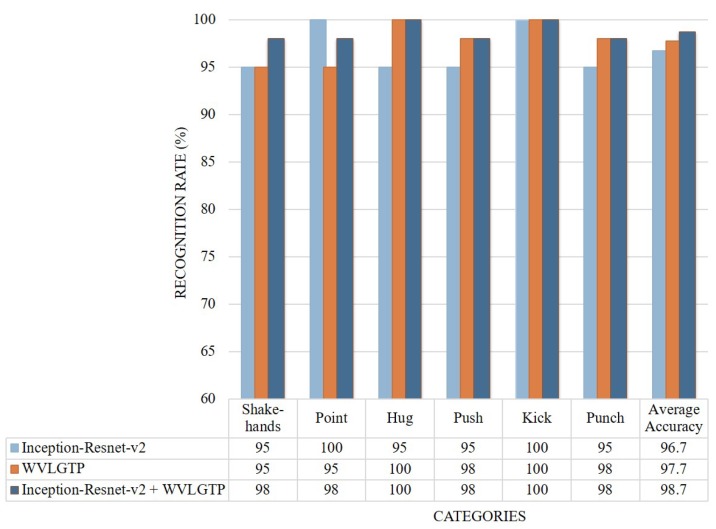
Accuracy comparison of each class by Inception-Resnet-v2, WVLGTP, and Inception-Resnet-v2 plus WVLGTP on the UT interaction dataset.

**Figure 13:**
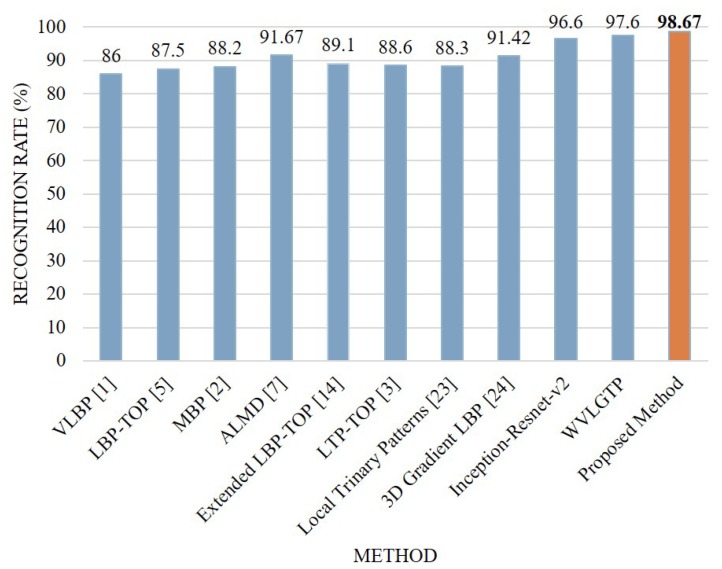
Comparison between the proposed method and other state-of-the-art approaches on the UT interaction dataset.

On the UT interaction dataset, the deep spatial features extracted through Inception-Resnet-v2 model shows 96.6% average accuracy while the proposed spatiotemporal feature descriptor WVLGTP shows 97.6% average accuracy. Since this dataset is more about the interaction rather than appearance information, due to this the proposed WVLGTP outperform the Inception-Resnet-v2 model. Figure 12 depicts the accuracy comparison of each class and Figure 13 presents the comparison among our work and other spatiotemporal feature descriptors on UT interaction dataset. From this experiment, we can see that the proposed approach outperforms the existing spatiotemporal feature descriptors by a large margin, which include outperforming VLBP [1], MBP [2], ALMD [7], and 3D Gradient LBP [24] by 12.67%, 10.47%, 7%, and 7.25%, respectively.

**Figure 14:**
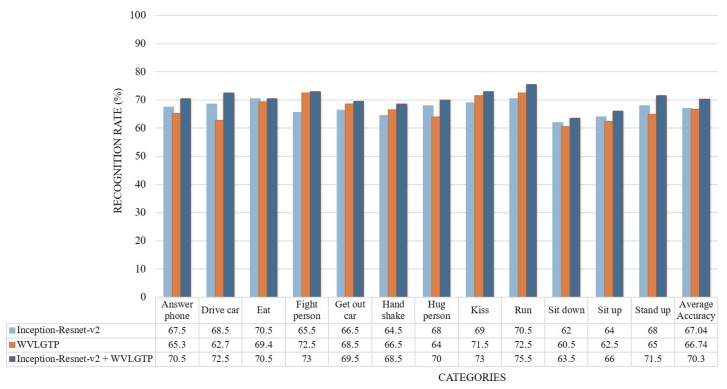
Accuracy comparison of each class by Inception-Resnet-v2, WVLGTP, and Inception-Resnet-v2 plus WVLGTP on the Hollywood2 dataset.

**Figure 15:**
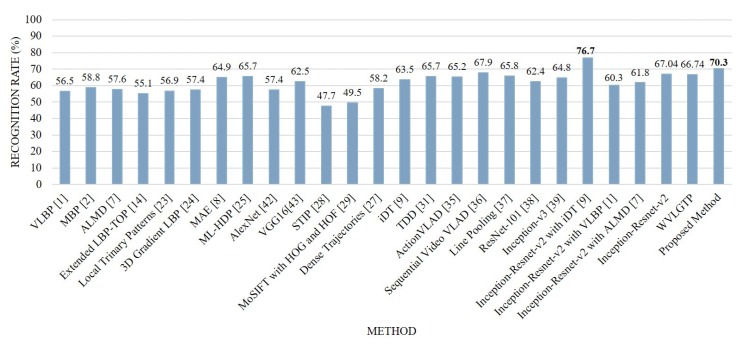
Comparison between the proposed method and other state-of-the-art approaches on the Hollywood2 dataset.

Figure 14 demonstrates the accuracy comparison of each class using the proposed approach while Figure 15 shows the comparison between our method and other state-of-the-art approaches on Hollywood2 dataset. In this experiment, run, kiss, and stand up categories shows the best performance while sit-down and sit-up shows the worst performance due to the similarities of these actions. The proposed spatiotemporal feature descriptor WVLGTP significantly outperformed the VLBP [1], MBP [2], ALMD [7], Extended LBP-TOP [14], 3D Gradient LBP [24], VGG [43] and AlexNet [42], while WVLGTP shows competitive performance with the MAE [8], ML-HDP [25], IDT [9], TDD [31], ActionVLAD [35], Line Pooling [37], ResNet-101 [38], and Inception-v3 [39] on Hollywoo2 dataset. In contrast, Sequential VLAD [36] shows better accuracy than the proposed WVLGTP. However, employing the proposed method by combining the deep spatial features extracted using Inception-Resnet-v2 and spatiotemporal features extracted using WVLGTP significantly outperforms the state-of-the-art approaches.

On Hollywood2 dataset, our proposed method (Inception-resnet-v2 with WVLGTP) shows 70.3% accuracy, while Inception-resnet-v2 with iDT [9], Inception-resnet-v2 with VLBP [1], and Inception-resnet-v2 with ALMD [7] show 76.7%, 60.3%, and 61.8% accuracy, respectively.

**Figure 16:**
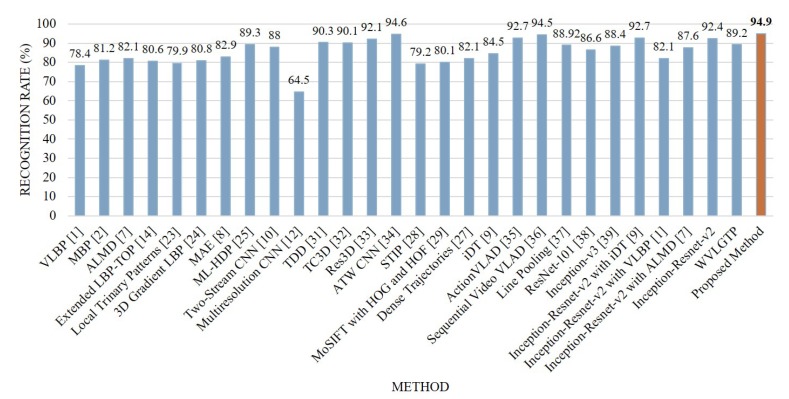
Comparison between the proposed method and other state-of-the-art approaches on the UCF101 dataset.

Lastly, Figure 16 shows the comparison between the proposed method and other state-of-the-art approaches on UCF101 dataset. From this experiment, we can see that the proposed feature descriptor, WVLGTP shows superior accuracy over the existing dynamic texture feature descriptors which includes VLBP [1], MBP [2], ALMD [7], and 3D Gradient LBP [24]. Similarly, WVLGTP shows competitive performance with Dense Trajectories [27], iDT [9], and Line Pooling [37]. However, TDD [31], Res3D [33], Action VLAD [35], and Sequential VLAD [36] show better accuracy than the proposed WVLGTP due to their discriminative power while employing a large number of action categories. On this dataset, the proposed method (Inception-Resnet-v2 plus WVLGTP) greatly outperforms the VLBP [1], MBP [2], ML-HDP [25], two-stream CNN [10], and multi-resolution CNN [12] by 16.5%, 13.7%, 5.6%, 6.9%, and 30.4%, respectively. In contrast, our approach slightly beats the TDD [31], TC3D [32], Res3D [33], ActionVLAD [35], and Sequential VLAD [36] since these approaches also achieved more discriminative power by considering the deep features and motion feature with CNN. Furthermore, ATW CNN [34] shows almost similar accuracy with our approach, since their approach incorporates the temporal attention with CNN.

For UCF-101 dataset, our proposed method (Inception-resnet-v2 with WVLGTP) shows 94.9% accuracy, whereas Inception-resnet-v2 with iDT [9], Inception-resnet-v2 with VLBP [1], and Inception-resnet-v2 with ALMD [7] shows 92.7%, 82.1%, and 87.6% accuracy, respectively.

**Figure 17:**
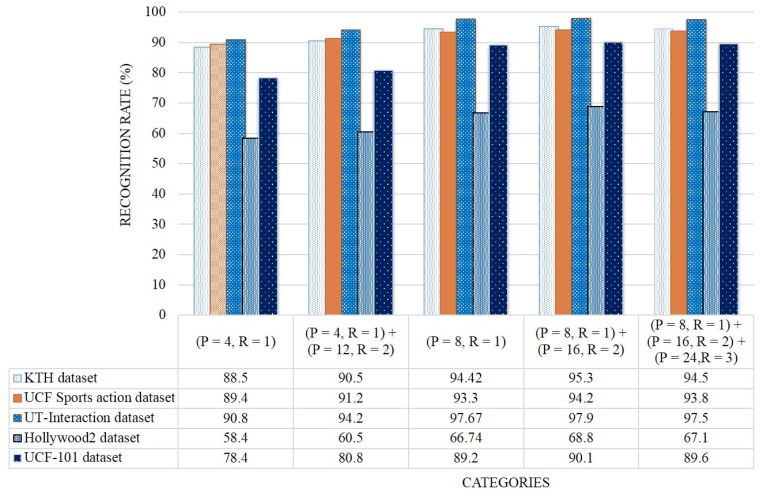
Classification accuracy (%) of WVLGTP with multi-resolution approach on all five datasets.

**Figure 18:**
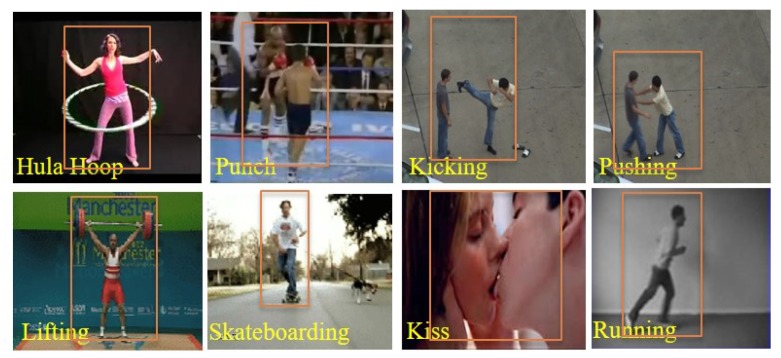
Some sample results of action recognition.

Figure 17 presents the average recognition rates of WVLGTP with multi-resolution approach on all five datasets. This experiment proves the effectiveness of multi-resolution approach. As can be seen in Figure 17, employing the number of neighboring pixels P=8 with radius R=1 and the number of neighboring pixels P=16 with radius R=2 yields the best performance, while applying the number of neighboring pixels P=4 with radius R=1 shows the worst performance due to less information. Furthermore, this experiment also indicates that the proposed WVLGTP consistently obtains the best recognition rates in almost all the multi-resolution environments. Finally, to validate the effects of the proposed method we visualize some sample results of action recognition on Figure 18. In summary, the experimental analysis proves the discriminative power of the proposed WVLGTP over existing spatiotemporal feature descriptor and moreover, it also proves the superiority of the proposed approach over state-of-the-art approaches.

## 5. Conclusions

In this paper, we combine both deep spatial features and handcrafted spatiotemporal features for action recognition. In order to obtain the deep spatial features from the video frames we adopt the state-of-the-art Inception-Resnet-v2 network, whereas the spatiotemporal features are computed by the proposed descriptor, called Weber’s law based Volume Local Gradient Ternary Pattern (WVLGTP). Finally, these features are concatenated and fed into an SVM for action classification. The experimental results prove the superiority of the proposed method over state-of-the-arts on five different datasets.
